# The Infection of Chicken Tracheal Epithelial Cells with a H6N1 Avian Influenza Virus

**DOI:** 10.1371/journal.pone.0018894

**Published:** 2011-05-06

**Authors:** Ching-I Shen, Ching-Ho Wang, Shih-Cheng Shen, Hsiu-Chin Lee, Jiunn-Wang Liao, Hong-Lin Su

**Affiliations:** 1 Department of Veterinary Medicine, National Chung-Hsing University, Taichung, Taiwan; 2 School of Veterinary Medicine, National Taiwan University, Taipei, Taiwan; 3 Department of Life Sciences, National Chung-Hsing University, Taichung, Taiwan; 4 Graduate Institute of Veterinary Pathobiology, National Chung-Hsing University, Taichung, Taiwan; 5 Department of Physical Therapy, China Medical University, Taichung, Taiwan; University of Minnesota, United States of America

## Abstract

Sialic acids (SAs) linked to galactose (Gal) in α2,3- and α2,6-configurations are the receptors for avian and human influenza viruses, respectively. We demonstrate that chicken tracheal ciliated cells express α2,3-linked SA, while goblet cells mainly express α2,6-linked SA. In addition, the plant lectin MAL-II, but not MAA/MAL-I, is bound to the surface of goblet cells, suggesting that SA2,3-linked oligosaccharides with Galβ1–3GalNAc subterminal residues are specifically present on the goblet cells. Moreover, both α2,3- and α2,6-linked SAs are detected on single tracheal basal cells. At a low multiplicity of infection (MOI) avian influenza virus H6N1 is exclusively detected in the ciliated cells, suggesting that the ciliated cell is the major target cell of the H6N1 virus. At a MOI of 1, ciliated, goblet and basal cells are all permissive to the AIV infection. This result clearly elucidates the receptor distribution for the avian influenza virus among chicken tracheal epithelial cells and illustrates a primary cell model for evaluating the cell tropisms of respiratory viruses in poultry.

## Introduction

Sialic acids (SAs), consisting of a core of nine-carbon monosaccharide, are usually linked to the outermost capping position of glycans that are conjugated to cell-surface glycoproteins or glycolipids [1]. Sialyltransferase adds SA to the terminal sugar residues, such as galactose (Gal), N-acetylglucosamine (GlcNAc) or N-acetylgalactosamine (GalNAc) [1]. The conjugation between Gal and SA can be either in the form of an α2,3 or an α2,6 glycosidic linkage. In mammals, N-acetylneuraminic acid (Neu5Ac) and N-glycolylneuraminic acid (Neu5Gc) are the two most common types of SA, but Neu5Ac is the major type of SA in birds [2].

Plant lectins extracted from *Maackia amurensis* (*M. amurensis* leukoagglutinin, MAL) and *Sambucus nigra* (*S. nigra* agglutinin, SNA) are usually applied for the detection of Neu5Acα2–3Gal (SAα2–3Gal) and SAα2–6Gal glycans in tissues, respectively [3]. Two types of *M. amurensis* lectins were discovered: one that can agglutinate erythrocytes (hemagglutinin) (MAH, also known as MAL-II) and one that can agglutinate leukocytes (MAL, also known as MAM, MAA, MAL-I). Although both MAL-I and MAL-II recognize the SAα2–3Gal glycan, previous studies [4] and recent glycan microarray data [5] demonstrated that subterminal sugars affect their binding affinity to these two lectins. For example, MAL-I, rather than MAL-II, showed the highest affinity to the SAα2–3Galβ1–4GalNAc and did not bind to this oligosaccharide when the subterminal β1,4-linkage was replaced by a β1,3-linkage [6].

Cell entry by influenza virus depends on the recognition of a terminal SA-capped glycosylated molecules by the viral hemagglutinin (HA) protein [3]. Generally, human influenza viruses preferentially bind to cell surface oligosaccharides that have the SAα2–6Gal linkage, while avian influenza viruses (AIVs) prefer SAα2–3Gal [3]. Especially, the glycans containing the SAα2–3Galβ1–4GalNAc or SAα2–3Galβ1–4(6OSO3)GalNAc, show a high affinity to MAA/MAL-I [4] and AIVs [7], [8]. Tracheal/bronchial epithelium, mainly consisting of ciliated cells, goblet cells and basal cells, is the initially attacked tissue by the invading influenza viruses. The infected chicken trachea shows necrosis and detachment of ciliated cells, suggesting that ciliated cell is one of the cells targeted by AIV [9]. Previous analyses of the expression of SAs in chicken have revealed that α2,3-linked SA was localized on tracheal ciliated cells and α2,6-linked SAs was also present in the tracheal tissues [10], [11], [12]. However, the detailed distribution of SAs among chicken tracheal epithelial (CTE) cells remains unclear. Clarification of the distribution and the expression intensity of influenza receptor on CTE cells will help us to understand the viral tropism, viral spreading and the pathogenesis of avian influenza viruses. Here, the SA distribution on CTE cells were identified by the staining of biotin-labeled plant lectins. A Taiwan-isolated AIV H6N1 was applied to characterize the cell tropism of AIV and the correlation of the cell tropism of AIV to the SA distribution on CTE cells was also explored.

## Results

### The SAα2–3Gal expression of primary CTE cells

**Figure 1:**
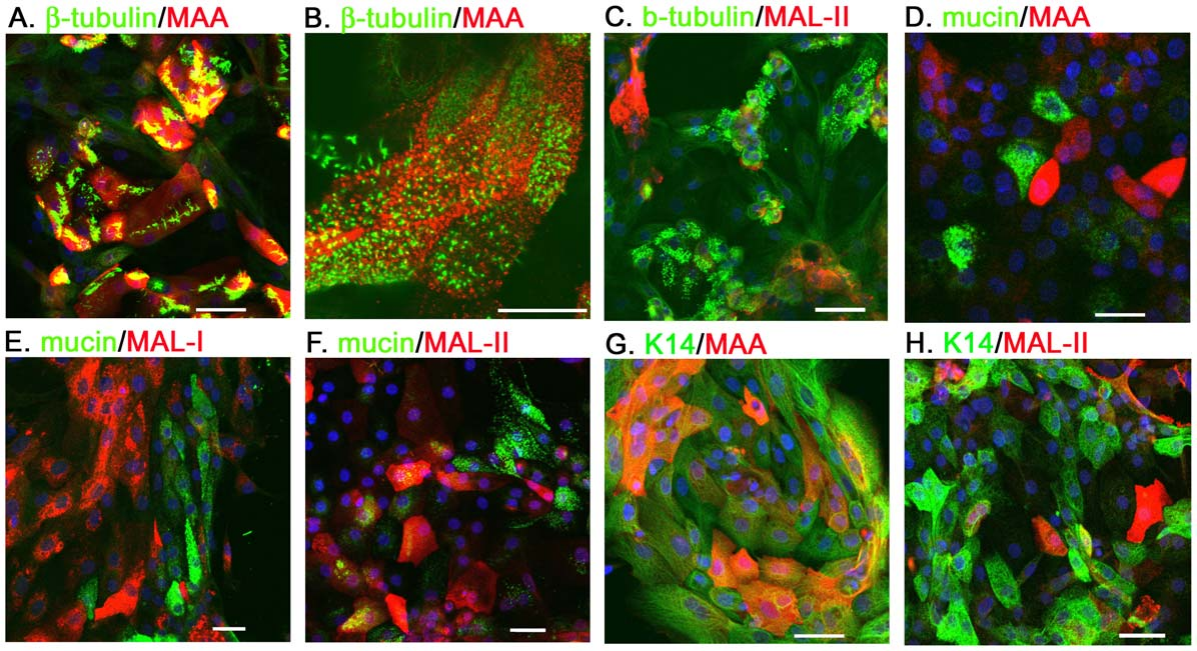
The SAα2–3Gal expression of primary CTE cells. The ciliated cells, goblet cells and basal cells were revealed by their specific markers: the β-tubulin (A–C), mucin (D–F) and K14 (G, H), respectively. The cellular distribution of SAα2–3Gal was characterized by the double-staining of biotin-labeled MAA (EY Laboratories, 1∶500) (A, B, D, G), MAL-1 (E) or MAL-II (both from Vector laboratories, 1∶500) (C, F, H). Scale bar, 50 µm.

The distribution of SAα2–3Gal expression on the primary CTE cells was determined by the staining with a plant lectin, MAA (EY Laboratories). The ciliated cells were revealed by the intense fluorescent microvilli, labeled by the β-tubulin specific antibody [13]. Mucin 5AC glycoprotein and cytokeratin-14 (K14) were the specific markers for the goblet and basal cells, respectively [14], [15]. Immunocytochemistry (ICC) staining illustrated that most β-tubulin^+^ cells expressed SAα2–3Gal terminal glycan (ratio of MAA^+^/β-tubulin^+^ cells, 0.87±0.06, n = 317) (Fig. 1A). In a high magnification field, the intense spots of MAA signal were surrounded by cilia bundles on the ciliated cells (Fig. 1B). In addition, MAA lectin could also be detected on the primary K14^+^ basal cells (ratio of MAA^+^/K14^+^ cells, 0.46±0.12, n = 267) (Fig. 1G).

Interestingly, the MAA signals did not colocalized with the mucin-expressing cells (ratio of MAA^+^/mucin^+^ cells, 0.007±0.01, n = 223) (Fig. 1D), indicating that goblet cells may not express the SAα2–3Gal glycans which show high affinity to the MAA lectin. However, for the immunohistochemistry in tissue sections, the distribution of SAα2–3Gal expression may show inconsistent distribution when different MAA/MALs were applied or the same lectins were from different providers [16], [17]. To further address the SA expression on goblet cells, MAL-I, same as MAA, was given by Vector Laboratories and applied to stain the CTE cells. We found that abundant MAL-I was detected on the β-tubulin^+^ cells (ratio of MAL-1^+^/β-tubulin^+^ cells, 0.82±0.10, n = 301) (Fig. S1), rather than the mucin^+^ cells (ratio of MAL-1^+^/mucin^+^ cells, 0.02±0.02, n = 201) (Fig. 1E), similar to the result for MAA.

Subtypes of SAα2–3Gal glycans with different subterminal sugars present varied affinity to MAA/MAL-I and MAL-II. Whether the SAα2–3Gal glycan is not expressed on goblet cells was further evaluated by MAL-II (Vector Laboratories). Surprisingly, 0.37±0.12 mucin^+^ cells (n = 594) were double-stained with MAL-II (Fig. 1F), indicating that a glycan subtype that shows a high affinity for MAL-II only, was expressed on the goblet cells. We also found that only 0.33±0.09 β-tubulin^+^ cells (n = 230) can be labeled with MAL-II (Fig. 1C). In addition, about half basal cells were MAL-II positive (0.53±0.12, n = 265) (Fig. 1H).

### The SAα2–6Gal expression of primary CTE cells

**Figure 2:**
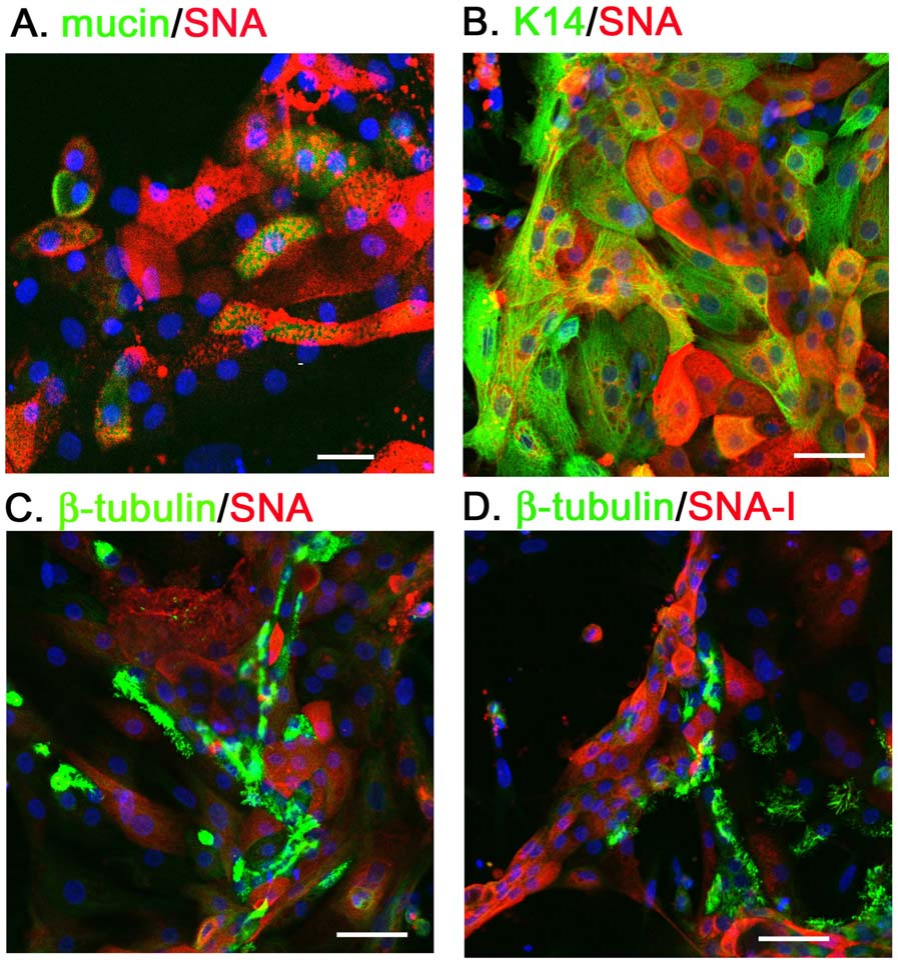
The SAα2–6Gal expression of primary CTE cells. The mucin (A), K14 (B) and β-tubulin (C and D) were the markers for goblet cells, basal cells and ciliated cells, respectively. The cellular distribution of SAα2–6Gal was shown by the double-staining of biotin-labeled SNA (Vector Laboratories, 1∶500) (A–C) and SNA-1 (EY laboratories, 1∶500) (D). Scale bar, 50 µm.

The distribution of SAα2–6Gal on the CTE cells was determined by staining with SNA (Vector Laboratories). In contrast to the finding for MAA/MAL-I, abundant SNA signals were mainly restricted to the mucin^+^ cells (ratio of SNA^+^/mucin^+^ cells, 0.85±0.09, n = 368) (Fig. 2A), indicating that the goblet cells expressed SAα2-6Gal terminal glycan. In addition, SNA lectin can also be detected in a one-third proportion of K14^+^ cells (ratio of SNA^+^/K14^+^ cells, 0.36±0.09, n = 284) (Fig. 2B).

Especially, the SNA signal did not localize on the β-tubulin^+^ cells (ratio of MAA^+^/β-tubulin^+^ cells, 0.01±0.01, n = 237) (Fig. 2C). Testing the SNA-I, which was provided by EY Laboratories, showed no difference to the result of SNA, evidenced by the undetectable binding by SNA-I on the ciliated cells (Fig. 2D).

### The SAα2–3Gal and SAα2–6Gal expression on basal cells

**Figure 3:**
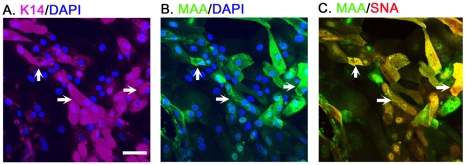
The SAα2–3Gal and SAα2–6Gal expression on basal cells. The tracheal basal cell was identified by the expression of K14 (1∶100, Convance) (A). Panels (A, B, C) with two fluorescent tags are shown in juxtaposition, illustrating the triple immunocytostaining result in a same field. The arrows in A, B and C indicate cells that are triple-positive for K14, MAA and SNA. The cell nuclei were stained with DAPI (blue). Scale bar, 50 µm.

Although both MAA and SNA bound to the surfaces of a subpopulation of the K14^+^ cells (Fig. 1G and Fig. 2B), their detected signals were relatively weak (Figs. S2A and S2B). To determine whether a single basal cells can express both SAα2–3Gal and SAα2–6Gal, CTE cells were triple-stained with K14 primary antibody (Cy5-2° Ab, purple), FITC-conjugated MAA (green) and biotin-labeled SNA (detected by Cy3-Streptovidin, red) (Figs. 3A, 3B and 3C). Their cell nuclei were revealed by DAPI staining (blue). When viewed in the same field under a confocal microscope, the ICC result indicated that MAA and SNA can be co-expressed on single K14^+^ cells (indicated by the arrows).

The distribution and relative expression levels of SAα2–3Gal and SAα2–6Gal are summarized in Table 1. It should be noted that because the relative affinities of the lectins to their targeted glycans may differ, comparing the intensity of staining between the MAA and SNA cannot be used as a quantitative comparison of the relative SAα2–3Gal and SAα2–6Gal expressions.

**Table 1:** The predominant glycans binding to the lectins and H6N1.

	Chicken tracheal epithelial cells			High affinity glycans
Cell Type	ciliated	goblet	basal	
**MAA/MAL-I**	87%^high^	<2%	46%^low^	**SAα2–3Galβ1–4(6OSO3)GlcNAcβ SAα2–3Galβ1–4GlcNAcβ**
**MAL-II**	33%^low^	37%^high^	53%^low^	**SAα2–3Galβ1–3(6OSO3)GalNAcα SAα2–3Galβ1–3(SAα2–6)GalNAcα**
**SNA**	<2%	85%^high^	36%^low^	**SAα2–6Galβ1–4GlcNAcβ**
**AIV H_6_N_1_ MOI = 0.1/1**	++/+++	−/+	−/++	

The value of percentage indicates the expressing ratio of cells; high or low indicates the expressing intensity in cells.

Symbols: +++, >70%; ++, 30 to 70%; +, 10 to 30%; −, <10%.

The glycan data are from Ref. [4] and Functional glycomics Gateway: http://www.functionalglycomics.org/static/index.shtml.

### The AIV H6N1 infection in CTE cells

**Figure 4:**
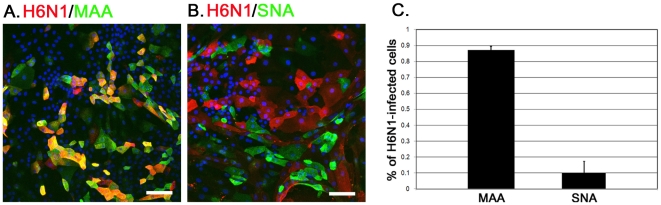
The SA expression on AIV H6N1 infected cells. A total of 5×10^4^ CTE cells were infected with 0.5 µl of AIV H6N1 2838V (viral stock, EID_50_ = 10^8^/ml) at a MOI of 1 for 1 h at 37°C. At 6 h.p.i., the expression of viral H6N1 proteins was detected by chicken serum against the AIV (1∶500, red) (A, B). The ratio of MAA or SNA expression on the infected cells was manually counted from five individual fields (C). Scale bar in panel A, 100 µm; in panel B, 50 µm.

This study used a Taiwan AIV H6N1 strain, 2838V (virulent), to determine the cell tropism of AIV. The CTE cells were infected with 2838V at a multiplicity of infection (MOI) of 1for 1 h in the DMEM medium and then the cells were cultured in the CTE culture medium at 37°C. At 6 h post-infection (h.p.i.), most of the infected cells were labeled with the MAA^+^ (0.87±0.02, n = 395) (Fig. 4A), but not with the SNA^+^ (0.10±0.07, n = 465) (Fig. 4B), indicating the preference of AIV H6N1 for the SAα2–3Gal receptor (Fig. 4C).

**Figure 5:**
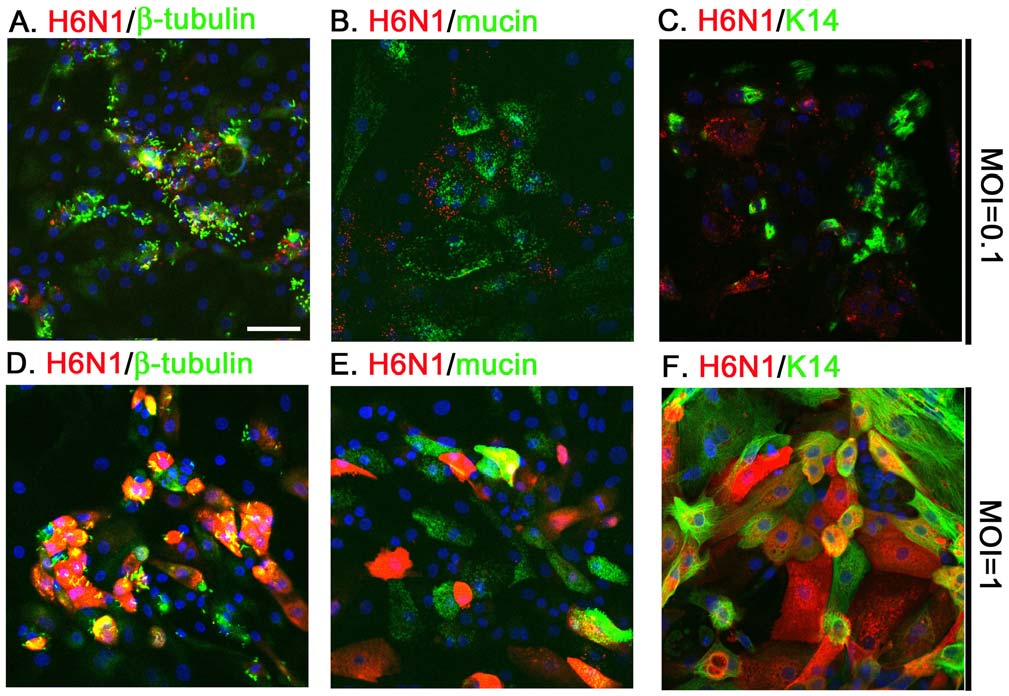
The tropism of AIV H6N1 for CTE cells. A total of 5×10^4^ CTE cells were infected with AIV H6N1 2838V at a MOI of 0.1 (A–C) or 1 (D–E) for 1 h at 37°C. At 6 h.p.i., infection by H6N1 was detected by chicken H6N1 immune-serum (1∶500, red). The ciliated cells, goblet cells and basal cells were revealed by the expression of β-tubulin (A, D), mucin (B, E) and K14 (C, F), respectively. Scale bar, 50 µm.

**Figure 6:**
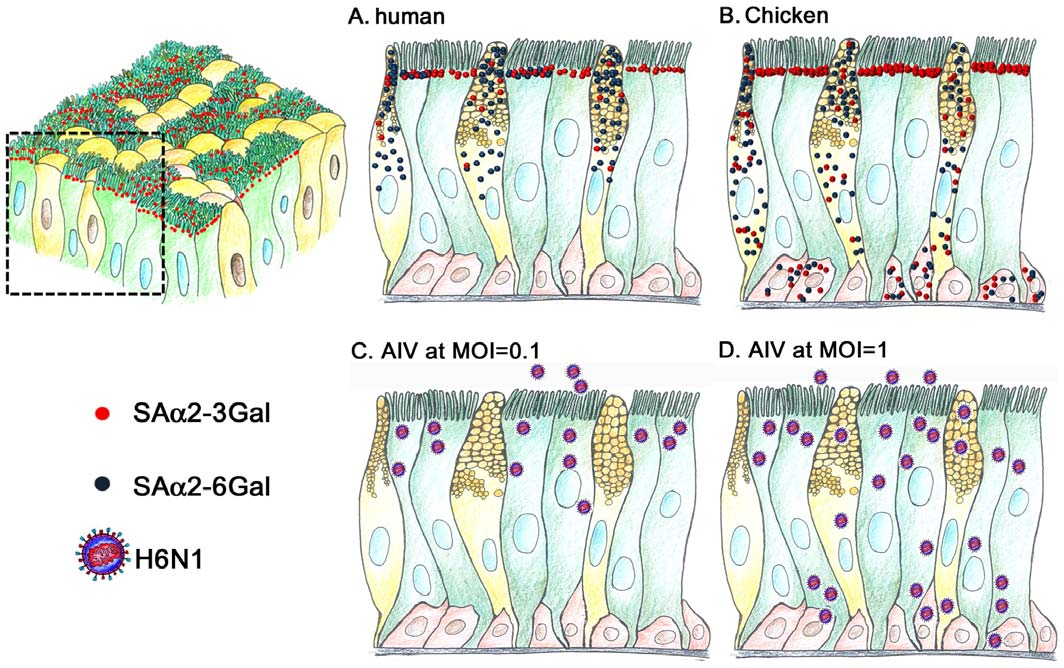
A speculated diagram of the SA expression and AIV infection in human and chicken tracheal epithelial cells. The structure of the pseudostratified tracheal epithelial cells (A) and the human (A) and chicken (B) expression profiles of SAα2–3Gal (red dots) and SAα2–6Gal (blue dots) on the ciliated cells (green), goblet cells (yellow) and basal cells (pink) are illustrated. Infection of chicken tracheal epithelial cells by AIV at a MOI of 0.1and 1 are illustrated in C and D, respectively.

In addition, the H6N1 viral antigens detected in the infected cells at a MOI of 0.1 were mostly restricted to the β-tubulin^+^ cells at 6 h.p.i. (Fig. 5A). Few H6N1 antigens were detected in the goblet cells or basal cells, possibly due to the low expression of AIV receptors (Figs. 5B and 5C). These results indicate that the ciliated cell, rather than the goblet or the basal cells, is the primary target cell for the AIV. In addition, these data also illustrate that the distribution of SAα2-3Gal expression among cells determines the cell tropism of the AIV H6N1 virus (as summarized in Fig. 6 and Table 1).

The infectivity of H6N1 in CTE cells was further characterized at a MOI of 1. ICC results showed that the ciliated cells (Fig. 5D), goblet and basal cells were all susceptible to infection by H6N1 as measured at 6 h.p.i. (Figs. 5E and 5F). Although SAα2–3Gal expression was relatively low in goblet and basal cells, still 21.3±0.05% of goblet cells and 51.1±10.9% of basal cells were infected. We speculated that the low infection of goblet cells by influenza virus at a high MOI might mediate through the binding to MAL-II specific SA or through a SA-independent pathway [18]. However, this hypothesis still requires the support from further experiments.

## Discussion

In the human tracheal/bronchial epithelium, ciliated cells display mainly α2,3- linked SAs and goblet cells express α2,6-linked SAs [19]. About one third of ciliated cells also display the α2,6-linked SAs and goblet cells also express the α2,3-linked SAs at a low degree [19], [20]. The distribution of the α2,3- and α2,6-linked glycans was primarily on the apical surface of both ciliated and goblet cells, respectively [19], [20]. As revealed by specific lectins and ICC staining in the primary CTE cells, our results clearly illustrate that all three types of CTE cells express SAα2-3Gal in a varied degree. SAα2-3Gal showed abundant expression on chicken ciliated cells but only a detectable level on basal cells (Fig. 6 and Table 1).

Revealing the distribution and the expression intensity of influenza virus receptor on the tracheal basal cells will help us to understand the cell tropism of influenza virus and the pathogenesis of AIV infected tissues. Our previous study indicated that the replication of infectious bronchitis virus (IBV) is restricted to ciliated cells and goblet cells, but not basal cells [21]. The tracheal/bronchial basal cells, assumed as multipotent stem cells, are responsible for epithelial recovery and reestablish normal respiratory function after desquamation of the ciliated and goblet cells [14], [15]. In an uncomplicated IBV-infected chick, clinical signs persist for only 1 week and the unaffected basal cells may be responsible for epithelial reconstruction of the injured respiratory tract [22]. In contrast, basal cells are susceptible to the infection of avian influenza virus at a MOI of 1. The susceptibility of basal cells to AIV infection suggests that infection with AIV alone may cause the cell death of basal cells, and consequently affect basal membrane integrity and severe inflammation in the AIV infected trachea.

Interestingly, MAA/MAL-1 did not bind to the surface of goblet cells, suggesting that certain SAα2–3Gal glycans that show high affinity for MAA/MAL-I lectins, such as SAα2–3Galβ1–4GlcNAcβ and SAα2–3Galβ1–4(6OSO3)GlcNAcβ [4], [5], are not present to a significant extent on the cell membranes of goblet cells. However, one-third of the goblet cells showed strong MAL-II staining, suggesting that the MAL-II specific glycans, such as SAα2–3Galβ1–3(SAα2–6)GalNAcα and SAα2–3Galβ1–3(6OSO3)GalNAcα are highly expressed on goblet cells [4], [5], [6], [23].

The expression profiles of SAs on CTE cells exhibits distributions that are distinct from those of human. In chickens, MAL-I bind to the surface of ciliated cells and basal cells, but not goblet cells. The goblet cells express a MAL-II specific SAα2–3Gal glycan. In addition, both SNA and SNA-I failed to label the β-tubulin^+^ cells, indicating that SAα2–6Gal is exclusively expressed on non-ciliated cells. In humans, by contrast, both ciliated and goblet cells can be labeled with MAA/MAL-I and SNA [19], [20], indicating that these two epithelial cells have both types of influenza viral receptors. Moreover, both ciliated and goblet cells cells are permissive to infection by human influenza viruses [19], [20]. Interestingly, using duck influenza A viruses to infect human airway epithelial cells, the viral antigen could only be detected in the ciliated cells, but not in the goblet cells, possibly due to the low SAα2–3Gal expression on the surface of goblet cells and a low MOI infection [19], [20].

Although the SAα2–6Gal glycans was detected in human tracheal/bronchial goblet cells [19], [20] and was capped on mucin protein [24], it was also proposed that tracheal mucin in the airway contains SAα2–3Gal [25] (Fig. 6A). Especially, this secreted mucin was shown to prevent infection by influenza viruses with a SAα2–3Gal preference in the airway [26]. This false-receptor effect may mask the HA of the AIV, disable the viral access to the goblet cells and also account, at least in part, for the hindrance of AIV infection to human goblet cell [26]. In chicken trachea, goblet cells exhibit high affinity for SNA, suggesting that the mucin may contain SAα2–6Gal glycans. The viral neutralization effect of mucin in humans may be recapitulated in chickens to aid the clearance of the invading influenza viruses with a SAα2–6Gal preference.

In addition to the SA2,6-linkage glycan, SA2,3-linked oligosaccharides with Galβ1–3GalNAc subterminal residues, which show a MAL-II preference, may be present in the goblet cells (Table 1) [4], [5], [6], [23]. Notably, although AIVs can bind to the terminal SAα2–3Gal, duck and chicken influenza viruses exhibit different affinities for glycans with different subterminal residues [7], [8]. For example, duck influenza viruses prefer Galβ1–3GlcNAc or Galβ1–3GalNAc, but chicken influenza viruses prefer Galβ1–4GlcNAc or Galβ1–4(6OSO3)GlcNAc [7], [8]. Our study suggests that SAα2,3-capped oligosaccharides following a Galβ1,3-linkage [6] are candidate sugars conjugated to the chicken tracheal mucin protein. Determining the SA composites of chicken mucin protein and the neutralizing effect of chicken mucin on duck influenza viruses will be an interesting task that will provide important information about the innate defense mechanisms in the chicken trachea and about the interspecies transmission of influenza viruses between ducks and chickens.

Both the hindrance of the mucin barrier and the limited number of proper receptors on the epithelial cells of the host restrict cross-species infection by influenza viruses [6], [20]. Nevertheless, genetic mutations or recombinations in the HA of an influenza virus can render interspecies transmission by altering the receptor tropism [27], [28], [29]. The 226 and 228 residues in the HA of the influenza H3 and H2 viruses are particular important for determining the receptor specificity and host range restrictions [30], [31]. The Ser-to-Gly mutation at HA position 228 and the Leu-to-Gln mutation at HA position 226 shift the receptor preference from SAα2–6Gal to SAα2–3Gal and enhance the infectivity of human influenza virus in duck intestinal cells [31].

The HA of the chicken H6N1 virus we applied possesses the GQRSRI sequence, which corresponds to the 225 to 230 positions in H3 HA [32]. We showed that most MAA^+^ cells, but few SNA^+^ cells, were permissive to infection by the H6N1 virus. In addition, at a low MOI, most H6N1 proteins were detected in the SAα2–3Gal-rich ciliated cells, and few in the goblet and basal cells. These results indicate that the chicken H6N1 virus, even with a Ser228 (using H3 HA numbering) in its HA, still possesses a SAα2–3Gal preference. Similarly, the HA 226 residue, critical to cell tropism in human H3 and H9N2, has been shown to be an insufficient binding site for determining the receptor tropism of the human H1, H2, H5 and the avian H6, H9 and H5N1 viruses [4], [33], [34], [35]. These results emphasize that receptor tropism is determined by several critical residues in the HA proteins, but these sites in HA are not highly conserved among different influenza viruses.

## Materials and Methods

### The AIV

A Taiwan AIV H_6_N_1_ strain, 2838V (virulent), was obtained by serial inoculation of an original field isolate, H_6_N_1_ 2838, into specific pathogen free (SPF) chickens [36]. The virus was further amplified by passage into the amnion of SPF eggs. Cell debris in the collected fluid was clarified by centrifuging at 1000 rpm for 10 min and passing through a 0.45 µm filter. The viral titer (50% of the egg infectious dose, EID_50_) of 2838V was 1×10^8^/ml.

### Primary culture of CTE cells

Tracheas were obtained from one-day-old SPF chicks (Animal Health Research Institute, Tansui, Taipei, Taiwan) and rinsed in a DMEM medium (Invitrogen, Carlsbad, CA, USA) under a sterile condition. The procedure for removing epithelial sheets from the tracheas and the detailed culture conditions for the CTE cells have been described previously [21]. Briefly, tracheas were digested with dispase I solution (2.5 U/ml dispase I, Roche) for 2 h at 37°C. The detached cell sheets of tracheal epithelium from the tracheal lumen were harvested and further digested with collagenase I (1 mg/ml, Roche) for 5 min at 37°C. The disrupted tracheal epithelial sheets were gently pippeted and homogenized into small cell clumps. The cell pellets were resuspended in a CTE medium [21] and were seeded on 2% matrigel-coated 24-well plates (Corning). The cells were cultured at 37°C with 5% CO_2_ for 3 days. The animal use protocol in this study had been reviewed and approved by Institutional Animal Care and Use Committee in National Chung Hsing University. The approval number is 99-09.

### Chemicals

The tested lectins for the SAα2–3Gal capped glycan were MAA (EY Laboratories, San Mateo, CA, USA), MAL-I (Vector Laboratories, Burlingame, CA, USA) and MAL-II (Vector Laboratories). The lectins for the SAα2–6Gal were SNA (Vector Laboratories) and SNA-I (EY Laboratories). The MAA lectins were conjugated with biotin or a green fluorescent dye, fluorescein isothiocyanate (FITC). The signal of the biotin-labeled lectins was enhanced and visualized by staining with a red-fluorescent Cy3-conjugated streptavidin (Rockland, Gilbertsville, PA, USA).

### Immunocytostaining

The CTE cells were fixed in 4% cold paraformaldehyde and blocked using the Carbo-free™ blocking solution (Vector Laboratories). Immunocytochemistry (ICC) was performed using the biotin-labeled lectins (MAA, MAL I, MAL II, SNA and SNA I, 1∶500) or the FITC-conjugated MAA (1∶50, EY Laboratories). The used primary antibodies were β-tubulin (1∶500, Sigma-Aldrich, St. Louis, MO, USA), mucin 5AC (1∶100, Sigma-Aldrich) and cytokeratin 14 (K14, 1∶100, Convance, Princeton, NJ, USA). The specificity of the anti-AIV 2838 polyclonal antibodies (1∶500, from infected chickens) has been evaluated in our previous report [37]. Appropriate fluorescence-tagged secondary antibodies (2° Ab, Jackson ImmunoResearch, West Grove, PA, USA) and Cy3-conjugated streptavidin (Rockland) were used for visualization. Blue 4′,6-Diamidino-2-phenylindole (DAPI) was used for nuclear staining. The number of ICC-staining positive cells in a 24-well plate was manually counted from five individual fields. Images of the immunostaining were captured using a confocal microscope (LSM510 Meta, Zeiss). For comparing the expression intensity of SA among CTE cells, the images were collected with the same setting and same objective (40×) under the confocal microscope.

## Supporting Information

Figure S1 **The SAα2–3Gal expression of ciliated cells (β-tubulin^+^) was characterized by the double-staining of biotin-labeled MAL-1 (Vector laboratories, 1∶500).** Scale bar, 50 µm.
(EPS)

Figure S2 **Expression levels of SAα2–3Gal (labeled by MAA)(A) and SAα2–6Gal (labeled by SNA)(B) in tracheal basal cell (K14^+^) were shown by immunocytostaining.** The arrows in A and B indicate cells with high-intensity MAA and SNA staining, respectively. The cell nuclei were stained with DAPI (blue). Scale bar, 50 µm.
(EPS)
